# Selection of RNA-based evaluation methods for tumor microenvironment by comparing with histochemical and flow cytometric analyses in gastric cancer

**DOI:** 10.1038/s41598-022-12610-w

**Published:** 2022-05-20

**Authors:** Noriyuki Saito, Yasuyoshi Sato, Hiroyuki Abe, Ikuo Wada, Yukari Kobayashi, Koji Nagaoka, Yoshihiro Kushihara, Tetsuo Ushiku, Yasuyuki Seto, Kazuhiro Kakimi

**Affiliations:** 1 Department of Gastrointestinal Surgery, The University of Tokyo Graduate School of Medicine, Tokyo, 113-8655 Japan; 2 Department of Immunotherapeutics, The University of Tokyo Hospital, 7-3-1 Hongo, Bunkyo-Ku, Tokyo, 113-8655 Japan; 3 Department of Medical Oncology, The Cancer Institute Hospital of Japanese Foundation for Cancer Research, Tokyo, 135-8550 Japan; 4 Department of Pathology, Graduate School of Medicine, The University of Tokyo, Tokyo, Japan; 5 Department of Surgery, Tokyo Metropolitan Bokutoh Hospital, Tokyo, 130-8575 Japan

**Keywords:** Tumour immunology, Gastrointestinal cancer, Tumour immunology, Cancer microenvironment

## Abstract

Understanding the tumor microenvironment (TME) and anti-tumor immune responses in gastric cancer are required for precision immune-oncology. Taking advantage of next-generation sequencing technology, the feasibility and reliability of transcriptome-based TME analysis were investigated. TME of 30 surgically resected gastric cancer tissues was analyzed by RNA-Seq, immunohistochemistry (IHC) and flow cytometry (FCM). RNA-Seq of bulk gastric cancer tissues was computationally analyzed to evaluate TME. Computationally analyzed immune cell composition was validated by comparison with cell densities established by IHC and FCM from the same tumor tissue. Immune cell infiltration and cellular function were also validated with IHC and FCM. Cell proliferation and cell death in the tumor as assessed by RNA-Seq and IHC were compared. Computational tools and gene set analysis for quantifying CD8^+^ T cells, regulatory T cells and B cells, T cell infiltration and functional status, and cell proliferation and cell death status yielded an excellent correlation with IHC and FCM data. Using these validated transcriptome-based analyses, the immunological status of gastric cancer could be classified into immune-rich and immune-poor subtypes. Transcriptome-based TME analysis is feasible and is valuable for further understanding the immunological status of gastric cancer.

## Introduction

The prognosis of locally advanced and metastatic gastric cancer remains poor, resulting in metastatic gastric cancers now being the fourth leading cause of cancer death globally^1^. Immune checkpoint inhibitors (ICIs) targeting the PD-1/PD-L1 pathway were approved for metastatic gastric cancer in 2017; this has improved the prognosis, but the benefits are nonetheless limited^2,3^. However, clinical indications for ICI are expanding, and different treatment strategies such as earlier administration or combinations with other chemotherapies are now becoming feasible^4,5^. To optimize these approaches, a rational design of combination immunotherapies for gastric cancer requires a better understanding of the tumor microenvironment (TME) and anti-tumor immune responses, which in turn requires optimal analytical methods for use in the clinic.

The Cancer Genome Atlas Research Network (TCGA) advocates comprehensive molecular characterization of gastric cancer based on RNA sequencing from bulk tissue (bulk RNA-Seq)^6^. Here, we undertook bulk RNA-Seq of gastric cancer and evaluated the TME based on the "Cancer-Immunity Cycle" that dynamically represents the intratumoral immune response^7^. We then proposed an immunogram classification for gastric cancer^8^. Although good therapeutic responsiveness to ICI has been shown in MSI-type and EBV-type gastric cancer^9^, responses to ICI in other types are poor. An immunosuppressive TME with complex heterogeneity might be one important reason for this difficulty in treating gastric cancer and remains to be further investigated.

RNA-Seq is an attractive tool for the interrogation of the transcriptome of a tumor and its microenvironment. It is possible to perform RNA-Seq analysis from either fresh-frozen or fresh tissue, with even a tiny piece of biopsy specimen sufficing. Compared to immunohistochemistry (IHC) and flow cytometry (FCM), many aspects of the immune response in the tumor can be investigated simultaneously with a very large number of markers by single method RNA-Seq. Although RNA-Seq provides comprehensive transcriptomic data, extracting biological insight and deducing the presence of different immune cells from such data requires computational analytical methods. However, there is a recognized problem regarding potential discrepancies between the quantification of cell populations based on transcriptomic data and the actual amount of the corresponding cell types estimated by IHC or FCM^10^. Therefore, further efforts to refine and improve the analysis of transcriptomic signatures are needed for their clinical application.

In this study, we examined the correlation between transcriptome-based analysis and IHC or FCM assessments, currently considered the gold standard for quantifying cell type composition and functional status. We selected the appropriate gene sets and computational analysis frameworks that reflect the TME of gastric cancer in terms of tumor-infiltrating lymphocytes, proliferation of tumor cells and immune cells, and tumor cell death. Using these validated computational methods, we propose a novel RNA-based evaluation of intratumoral immune responses in gastric cancer.

## Results

### Patients’ characteristics

The characteristics of patients in the BKT cohort are shown in Table 1 and Supplementary Table S1. Five of 30 patients had Stage IV disease and underwent palliative surgery. The HER2-positive rate was 23.3%, and the percentage of each TCGA molecular classification was as follows: MSI (16.7%), EBV (10.0%), GS (13.3%), and CIN (60.0%). Thus, the cohort appears to be representative, with no significant deviations from previous reports^6,11^.

**Table 1:** Characteristics of individual patients.

Baseline clinicopathological characteristics			
Age (years), mean ± SD	72.8 ± 9.1	**Histology (differentiation), n (%)**	
**Sex, n (%)**		Differentiated	16 (53.3)
Male	26 (86.7)	Undifferentiated	14 (46.7)
Female	4 (13.3)	Histology (Lauren classification), n (%)	
**Locus, n (%)**		Intestinal	19 (63.3)
GE	2 (6.7)	Diffuse	1 (3.3)
U	9 (30.0)	Mixed	9 (30.0)
M	8 (26.7)	Indeterminant	1 (3.3)
L	11 (36.7)	**HER2 status, n (%)**	
**Macroscopic type (Borrmann), n (%)**		Positive	7 (23.3)
1	2 (6.7)	Negative	21 (70.0)
2	14 (46.7)	Unknown	2 (6.7)
3	11 (36.7)	***Helicobacter pylori* infection, n (%)**	
4	2 (6.7)	Positive	20 (66.7)
5	1 (3.3)	Negative	10 (33.3)
**pStage, n (%)**		**Molecular classification by TCGA, n (%)**	
I	4 (13.3)	MSI	5 (16.7)
II	7 (23.3)	EBV	3 (10.0)
III	14 (46.7)	GS	4 (13.3)
IV	5 (16.7)	CIN	18 (60.0)
		**Mesenchymal subtype by ACRG, n (%)**	
		Mesenchymal	6 (20.0)
		Non-mesenchymal	24 (80.0)

### Evaluation of transcriptome-based cell type quantification

The different transcriptome-based immune cell quantification methods listed in Table 2 were applied to RNA-Seq data of 30 gastric cancer patients (Supplementary Table S2). Multiple immune cells were computationally quantified from one RNA-Seq dataset. These methods can be divided into two categories, namely, values for the expression of marker genes (marker-based approach) or the deconvolution approach. The output scores are the absolute value of each cell population or the fraction (%) of the total cells. Therefore, certain defined cell type data can be compared between patients but cannot be compared to other cell types in the same patient (i.e. inter-sample comparisons of the same cell type but not intra-individual comparisons of different cell types). On the other hand, some cell population data can be compared to other cell populations within the analyzed patient but not the same cell population in a different patient (i.e. intra-sample comparison between cell types). To validate these transcriptome-based data (Supplementary Table S2), they were compared to IHC or FCM data. Formalin-fixed paraffin-embedded (FFPE) slides were stained with the indicated antibodies (Supplementary Fig. S1). Images were digitally captured and analyzed with Tissue Studio 2.0 to establish immune cell numbers and calculate the immune cell densities (/mm^2^) (Supplementary Table S3). Tumor-infiltrating immune cells were also evaluated by FCM (Supplementary Table S4).

**Table 2:** Transcriptome-based immune cell quantification methods.

Tool	Approach	Method	Output score	Cell types	Comparisons	References	On-line information
MCP-counter	Marker-based	Mean of marker gene expression	Arbitrary units, comparable between samples	8 immune cells, fibroblast, endothelial cell	Inter	Becht et al. Genome Biology (2016)	http://134.157.229.105:3838/webMCP/
xCell	Marker-based	ssGSEA	Arbitrary units, comparable between samples	34 immune cells, 9 other haematopoietic and 21 non-haematopoietic lineage cells	Inter	Aran et al. Genome Biology (2017)	https://xcell.ucsf.edu/
Bindea	Marker-based	ssGSEA	Arbitrary units, comparable between samples	24 immune cells, angiogenesis & antigen presentation machinery	Inter	Bindea et al. Immunity (2013)	https://cloud.genepattern.org/gp/pages/index.jsf
Davoli	Marker-based	ssGSEA	Arbitrary units, comparable between samples	10 immune cells	Inter	Davoli et al. Science (2017)	https://cloud.genepattern.org/gp/pages/index.jsf
Danaher	Marker-based	ssGSEA	Arbitrary units, comparable between samples	14 immune cells	Inter	Danaher et al. J Immunother Cancer (2017)	https://cloud.genepattern.org/gp/pages/index.jsf
ConsensusTME	Marker-based	ssGSEA	Arbitrary units, comparable between samples	16 immune cells, fibroblast, endothelial cell	Inter	Jiménez et al. Cancer Res (2019)	https://cloud.genepattern.org/gp/pages/index.jsf
TIMER	Deconvolution	Linear least square regression	Arbitrary units, comparable between samples (not different cancer types)	6 immune cells	Inter	Li et al. Genome Biol (2016)	http://timer.cistrome.org/
quanTlseq	Deconvolution	Constrained least square regression	Cell fractions, relative to all cells in sample	10 immune cells	Inter, Intra	Finotello et al. Genome Med (2019)	https://icbi.i-med.ac.at/software/quantiseq/doc/
EPIC	Deconvolution	Constrained least square regression	Cell fractions, relative to all cells in sample	6 immune cells, fibroblast, endothelial cell, uncharacterized cell type	Inter, Intra	Racle et al. Elife (2017)	http://epic.gfellerlab.org
CIBERSORTx (absolute)	Deconvolution	Support vector regression	Score of arbitrary units that reflects the absolute proportion of each cell type	22 immune cells	Inter, Intra	Newman et al. Nat Biotechnol (2019)	https://cibersortx.stanford.edu/
CIBERSORTx (relative)	Deconvolution	Support vector regression	Immune cell fractions, relative to total immune cell content	22 immune cells	Intra	Newman et al. Nat Biotechnol (2019)	https://cibersortx.stanford.edu/

*ssGSEA* single-sample gene set enrichment analysis.

**Figure 1:**
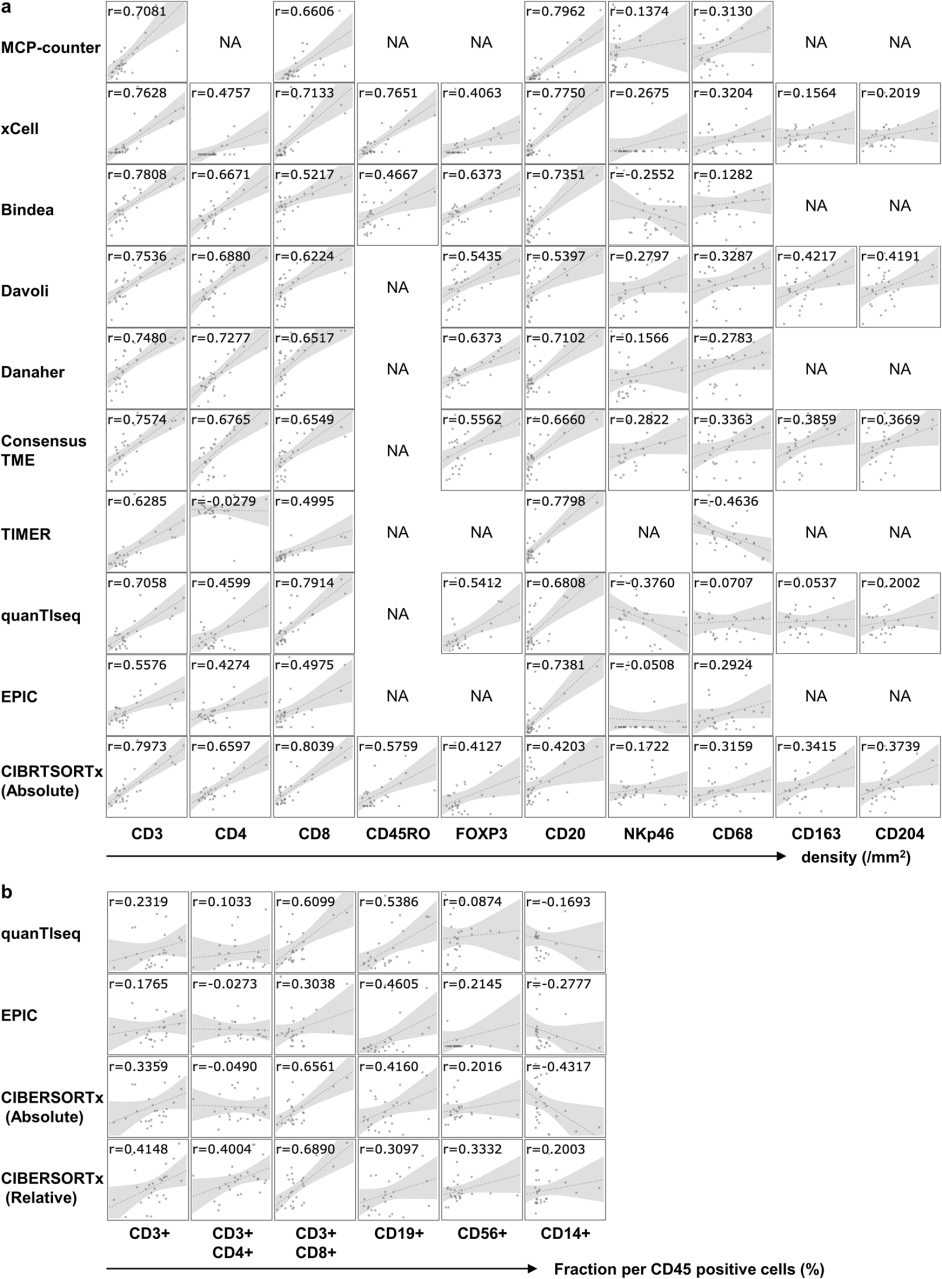
Correlation between transcriptome-based quantification methods and IHC or FCM. The vertical axis shows arbitrary scores obtained by the indicated computational method for RNA-Seq. (**a**) The horizontal axis shows the densities of immune cells detected by the indicated antibodies (/mm^2^). (**b**) The horizontal axis shows the percentages of the indicated antibody-positive cells in the sample as assessed by FCM. The Pearson's correlation coefficient (r) between the two is shown in the upper part of each scatter plot. Transcriptome-based cell quantification methods for inter-sample comparison were validated with IHC (**a**) and intra-sample comparison with FCM (**b**).

Figure 1 summarizes the results of bivariate Pearson correlations between the indicated computational transcriptome-based quantification methods and IHC/FCM data for the corresponding cells. The estimated scores of T cells and B cells by the RNA-Seq-based methods correlated closely with the density of CD3-, CD4-, CD8-, and CD20-positive cells by IHC (Fig. 1a) and the fraction of CD3-, CD8-, CD19- positive cells by FCM (Fig. 1b). Among them, the estimation of CD8-positive T cells in CIBERSORTx absolute mode exhibited the highest coefficient (0.8039). In addition, the single-sample Gene Set Enrichment Analysis (ssGSEA) score using the gene set of Bindea et al. and Danaher et al. for FOXP3-positive regulatory T cells (0.6373) and the MCP-counter for CD20-positive B cells (0.7962) also showed a high correlation coefficient with IHC. On the other hand, transcriptome-based quantification of NK cells (NKp46) or macrophages (CD68) displayed less correlation with IHC; the highest correlation coefficient with NK cells (NKp46) and macrophages (CD68) was obtained by quanTIseq (0.3760) and TIMER (0.4636), respectively. For transcriptome-based estimation, PBMC benchmarks may be less informative^12^. Therefore, we performed ssGSEA analysis using the gene set for tumor-associated macrophages (TAM) by Cassetta et al.^13^. However, it could not improve the correlation with CD68, CD163 or CD204-positive cells (0.4148, 0.4708 or 0.4119, respectively, Supplementary Fig. S2). In Supplementary Fig. S3, the transcriptome-based methods were ranked according to the correlation coefficient with the densities of immune cells. None of the methods outperformed others in every immune cell type.

### Spatial and functional analysis of tumor-infiltrating T cells

**Figure 2:**
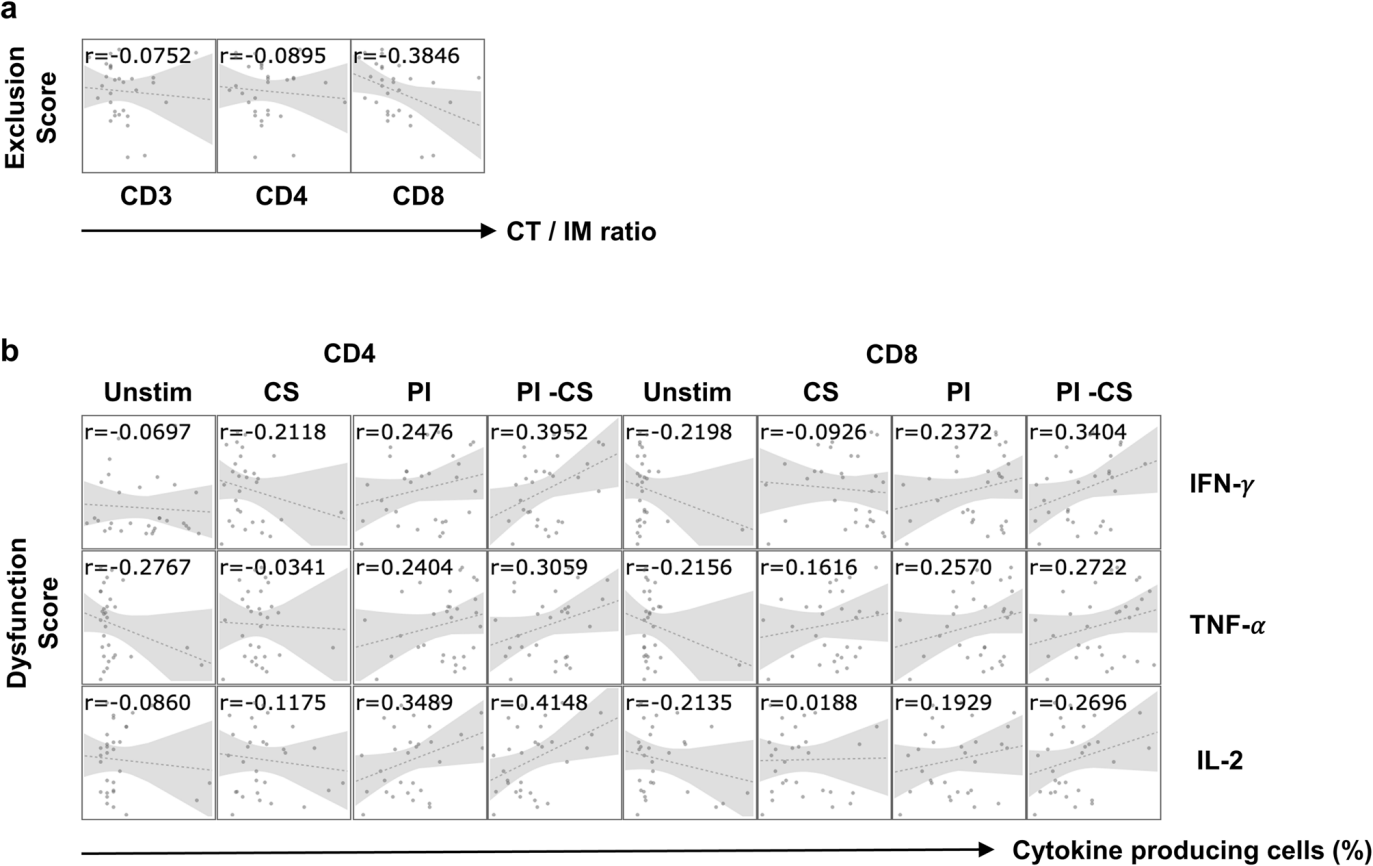
Correlations between TIDE scores and IHC or FCM. T cell dysfunction and exclusion scores were calculated on the TIDE website (http://tide.dfci.harvard.edu/). (**a**) The vertical axis shows the exclusion score. The horizontal axis shows the ratio of cell densities at CT versus IM of the following antibody-positive cells in IHC; CD3, CD4 and CD8. (**b**) The vertical axis shows the dysfunction score. The horizontal axis shows the percentage of cytokine (IFN-γ, TNF-α, IL-2)-producing CD4^+^ or CD8^+^ T cells without stimulation (Unstim), with CytoStim stimulation (CS), or PMA/IM (PI) stimulation, as detected by FCM. In addition, the difference between cytokine-positive cells with PI- and CS-stimulated cells was calculated in each patient and compared (PI-CS). The Pearson's correlation coefficient (r) between dysfunction scores and the percentages of cytokine-producing cells is shown in the upper part of each scatter plot.

Compared to IHC, spatial analysis by RNA-Seq is challenging. First, immune cell densities at the core of the tumor (CT) and invasive margin (IM) of the tumor were enumerated in each patient (Supplementary Fig. S4 and Supplementary Table S5). Next, RNA-Seq data were subjected to Tumor Immune Dysfunction and Exclusion (TIDE) framework approaches to compute a T cell exclusion score (Supplementary Table S6)^14^. Finally, the exclusion scores of 30 gastric cancer patients were compared to the ratio of cell densities at the CT versus IM of the CD3^+^, CD4^+^ or CD8^+^ cells (Fig. 2a). TIDE exclusion scores negatively correlated with the ratio of CT/IM densities for CD8^+^ cells, suggesting that RNA-Seq can be utilized to evaluate the spatial distribution of immune cells, particularly CD8^+^ T cells.

Tumor-infiltrating cells (TICs) were isolated from the surgically resected tumors and their capacity to produce IFN-γ, TNF-α and IL-2 was examined by FCM (Supplementary Fig. 5a and Table S7). TICs were left unstimulated or were stimulated with CytoStim (CS), which stimulates T cells via the T cell receptor (TCR), or were stimulated with Phorbol 12-Myristate 13-Acetate/Ionomycin (PMA/IM; PI), which directly increases intracellular calcium concentration without TCR signaling and results in cytokine expression (Supplementary Fig. S5b). Were TCR signaling to be suppressed by the immune inhibitory molecules, cytokine production following CytoStim stimulation would be decreased relative to PMA/IM stimulation. Therefore, using FCM, the level of T cell dysfunction can be evaluated by the differences in the percentage of cytokine-producing cells between PMA/IM stimulation versus CytoStim stimulation (PI-CS). As shown in Fig. 2b, TIDE dysfunction scores of the 30 patients correlated well with differences in the percentages of cytokine-producing cells of PI-CS (Fig. 2b).

### Proliferation of tumor cells and immune cells

**Figure 3:**
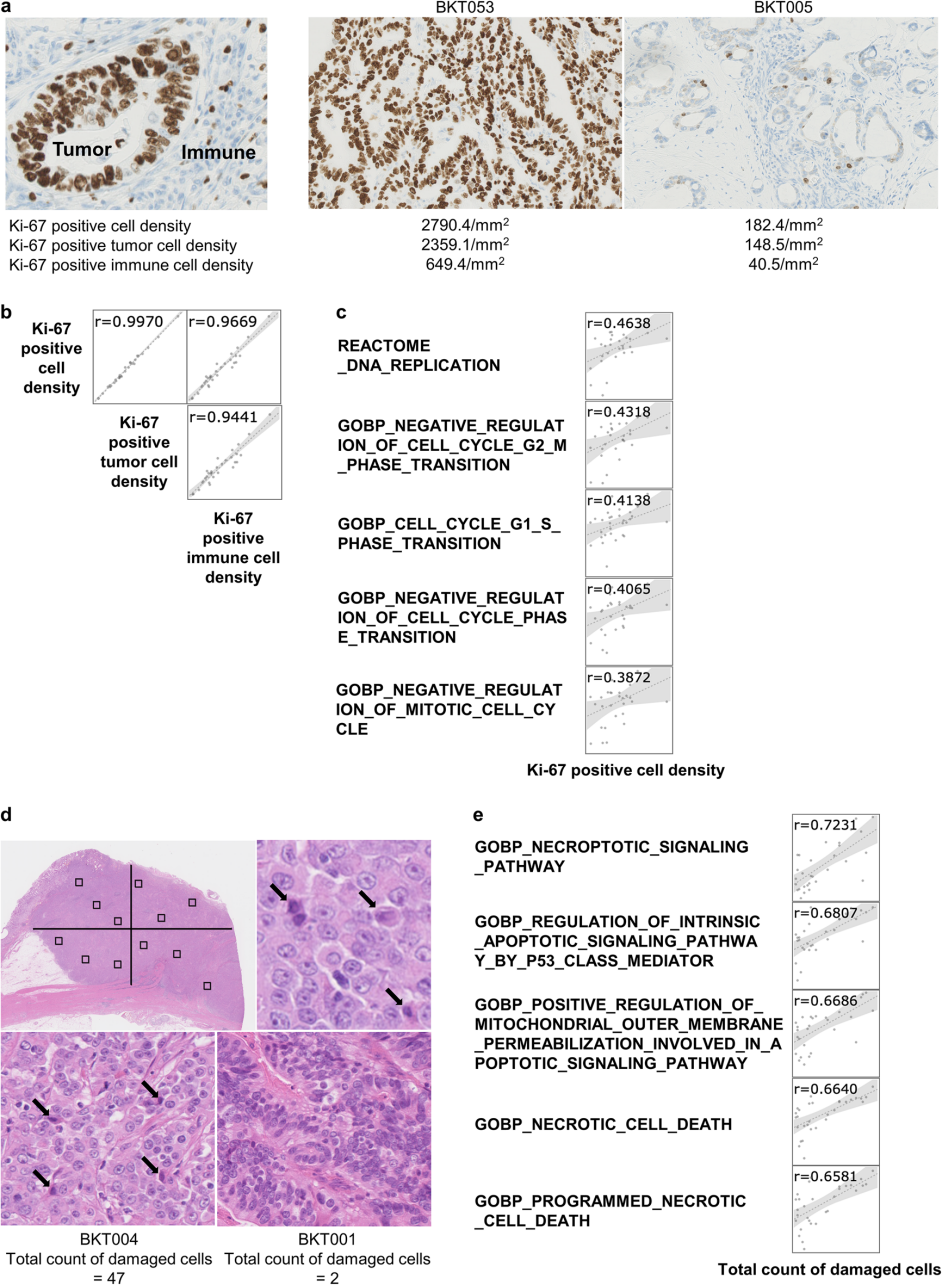
Cell proliferation and cell death in the tumor. (**a**) The slides were stained with anti-Ki-67 and the number of Ki-67^+^ cells was counted in the whole tumor area. The Ki-67^+^ cell densities were calculated as the number of Ki-67^+^ cells divided by tumor tissue area (mm^2^). Cells with a nucleus size ≥ 30 μm^2^ were considered to be cancer cells, and those with < 30 μm^2^ were regarded as immune cells. Slides with the most (BKT053) and the least (BKT005) Ki-67^+^ cells are shown. (**b**) Correlations between scores for the indicated Ki-67^+^ cell densities were examined. The Pearson's correlation coefficients (r) between the two are indicated at the top of the panels. (**c**) The vertical axis shows the indicated ssGSEA scores. The horizontal axis shows the Ki-67^+^ cell densities. (**d**) In H&E slides, tumor cells with elevated cytoplasmic acidity, nuclear fragmentation, or enrichment were defined as damaged cells (arrow). The tumor tissue was equally divided into 4 parts, and 3 areas of 25 mm^2^ each were randomly selected in each fraction. The number of damaged cells was counted in each area and the total number of damaged cells was obtained as the sum of the numbers from 12 areas. The tissues with the most (BKT004) and the least (BKT001) damaged cells are indicated. (**e**) The vertical axis shows the indicated ssGSEA scores. The horizontal axis shows the number of damaged cells. Correlations between the ssGSEA score for cell death and the total number of damaged cells in H&E slides were examined. The Pearson's correlation efficient (r) is shown in the upper part of each scatter plot.

Ki-67 staining is a well-established method for detecting proliferating cells. In the case of IHC, Ki-67^+^ tumor cells and immune cells can be discriminated morphologically (Fig. 3a, Supplementary Table S8). However, RNA-based evaluation methods for cell proliferation cannot predict the type of proliferating cells. As shown in Fig. 3b, the densities of Ki-67^+^ tumor cells, Ki-67^+^ immune cells and all Ki-67^+^ cells (both tumor and immune cells) were closely correlated with one another (Fig. 3b). These results suggest that extensively proliferating tumor cells are associated with proliferating immune cells. Therefore, we screened the appropriate gene sets to reflect the proliferation of the tumor cells and the immune cells all together (Supplementary Table S9). The ssGSEA scores of DNA REPLICATION from the REACTOME subset of canonical pathways in MSigDB (http://www.gsea-msigdb.org/gsea/msigdb/collections.jsp) displayed the highest correlation with the Ki-67-positive cell density by IHC (Fig. 3c).

### Cytotoxic activity in the tumor

As shown in Fig. 3d, damaged tumor cells were detected and enumerated on histology slides (Supplementary Table S10). The correlation between the total cell death count by histology and ssGSEA scores was examined to identify the appropriate gene sets for evaluating cell death in the tumor (Supplementary Table S11). GOBP_NECROPTOTIC_SIGNALING_PATHWAY from MSigDB exhibited the highest correlation coefficient of 0.7231 (Fig. 3e).

### TME analysis with the reliable gene sets

To obtain cell fractions that can be compared within samples (intra-sample comparison), quanTIseq and CIBERSORTx are recommended. For inter-sample comparison of any cells of interest, we selected 7 reliable transcriptome-based parameters supported by histology and FCM to evaluate the gastric cancer TME (Table 3 and Supplementary Fig. S3). In terms of the numbers and percentages of immune cells, the CIBERSORTx absolute mode for CD8^+^ T cells, Bindea's and Danaher’s gene set analysis for regulatory T cells, and the MCP-counter for B cells were selected. IHC and FCM confirmed the appropriateness of using the TIDE framework for evaluating the exclusion and dysfunction of immune cells in gastric cancer. The ssGSEA scores of REACTOME_DNA_REPLICATION and GOBP_NECROPTOTIC_SIGNALING_PATHWAY can be utilized for evaluating proliferation and cell death in the tumor.

**Table 3:** Validated transcriptome-based analysis.

Category	Selected bulk RNA-Seq analysis
(1) Immune cell estimation	1_CIBERSORTx (absolute)_T cells CD8
	2_Bindea_Treg
	3_MCP-counter_B lineage
(2) Infiltration and function	4_TIDE_Exclusion
	5_TIDE_Dysfunction
(3) Proliferation	6_ssGSEA_REACTOME_DNA_REPLICATION
(4) Tumor cell death	7_ssGSEA_GOBP_NECROPTOTIC_SIGNALING_PATHWAY

**Figure 4:**
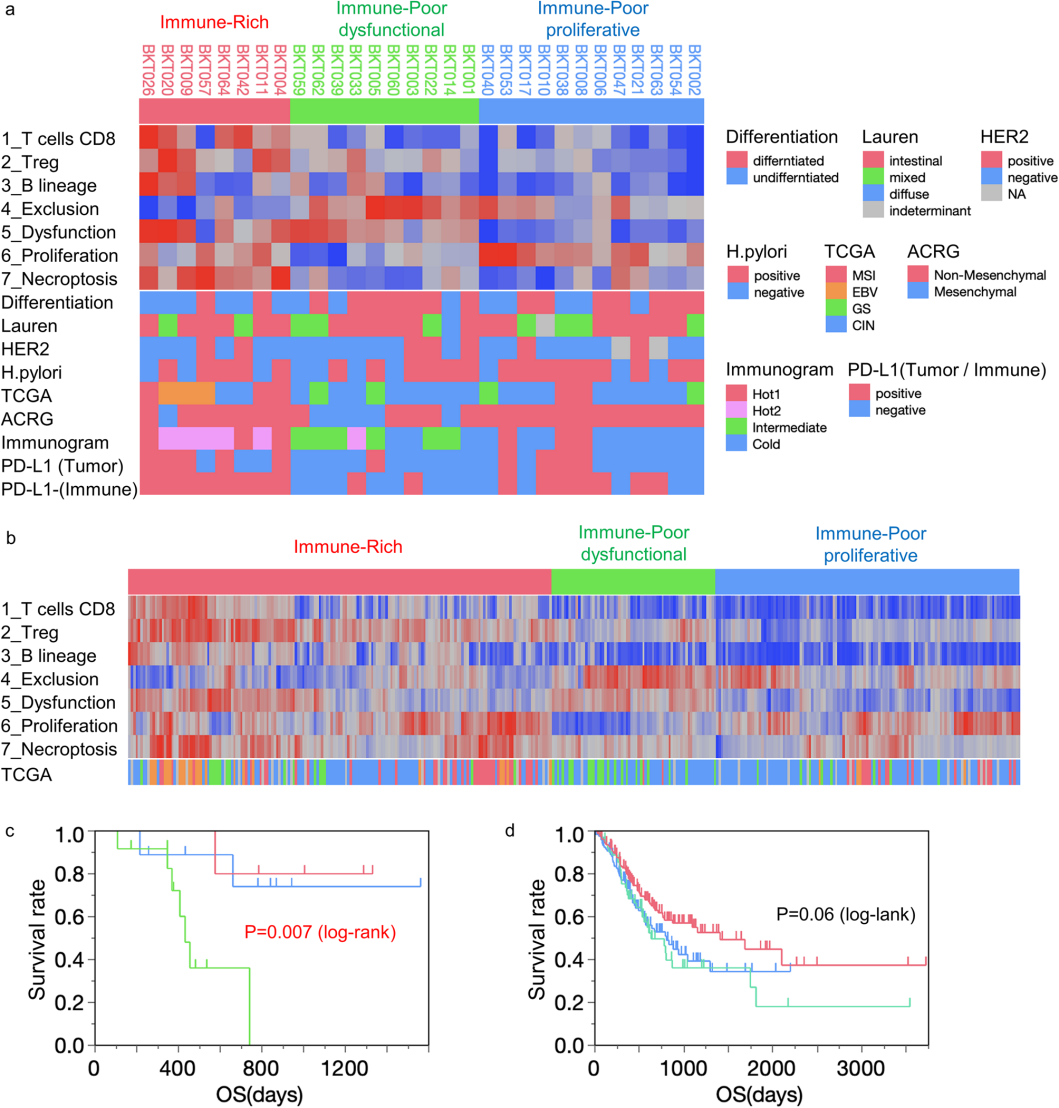
Transcriptome-based TME analysis with selected reliable gene sets in gastric cancer. (**a**) Hierarchical cluster analysis was performed in our 30 gastric cancer patients (BKT Cohort). The patients’ characteristics are shown in Table 1 and Supplementary Table S1. Degree of tumor differentiation, Lauren classification, HER2 status, *Helicobacter pylori* infection, TCGA subtype, the Asian Cancer Research Group (ACRG) Mesenchymal subtype^15^, Sato's immunogram classification (IGS)^8^ and PD-L1 IHC are displayed at the bottom. (**b**) Gastric cancer patients from the TCGA cohort (n = 375) were subjected to hierarchical clustering with 7 transcriptome-based TME analyses. The patients’ characteristics are shown in Tables S12-1 and S12-2. The molecular classification of TCGA is indicated at the bottom. Survival analysis for Immune-Rich (IR, red), Immune-Poor dysfunctional (IPd, green) and Immune-Poor proliferative (IPp, blue) groups. The Kaplan–Meier method and log-rank test were performed in BKT cohort (**c**) and TCGA cohort (**d**).

With these 7 selected transcriptome-based methods, the inter-sample comparison of TME in 30 gastric cancer patients was performed. The 30 gastric cancer patients were first clustered into two groups; the Immune-Rich (IR) and the Immune-Poor (IP) groups (Fig. 4a). Scores for CD8^+^ T cells, Tregs and B cells were high in the IR group, whereas the exclusion scores were consistently low. T cells became dysfunctional and both proliferation and cell death were evident in the IR patients. The IP group was further divided into IP dysfunctional (IPd) and proliferative (IPp). Scores for Tregs, exclusion, and dysfunction were high in IPd, while scores for proliferation were high and dysfunction were low in IPp. Patients with MSI and EBV subtypes (TCGA molecular classification) or “Hot” tumors (by Sato's immunogram classification^8^) were enriched in the IR group, while GS and CIN types were enriched in the IP group. In addition, PD-L1 expression by tumor or immune cells was present in IR patients. In survival analysis, IPd patients had the worst overall survival (OS) (Fig. 4c, *P* = 0.007, log-rank test).

Three hundred seventy-five gastric cancer patients from the TCGA cohort were similarly clustered into IR, IPd and IPp groups (Fig. 4b and Supplementary Table S12). Although the differences were not statistically significant, the worst OS in the IPd group was also detected (Fig. 4d, *P* = 0.06, log-rank test). We compared the survival analysis of the TCGA cohort between Sato’s immunogram classification^8^ and the transcriptome-based TME classification of this study. Eighty Hot1 cases were re-classified as 61 IR (Hot1-IR), 10 IPd (Hot1-IPd) and 9 IPp (Hot1-IPp) subtypes (Supplementary Fig. S6a). OS of Hot1-IR was better than that of Hot1, and OS of Hot1-IPd and Hot1-IPp was worse than that of Hot1 (Supplementary Fig. S6b). Similarly, 187 Cold cases were classified as 34 IR (Cold-IR), 38 IPd (Cold-IPd) and 115 IPp (Cold-IPp). OS of Cold-IR was better than that of Cold, while OS of Cold-IPd and Cold-IPp was comparable to that of Cold patients (Supplementary Fig. S6e).

## Discussion

RNA-Seq data of bulk tumor tissues treats heterogeneous cell populations as a whole; data are averages of different cells with various gene expression levels. Therefore, computational methods to evaluate cellular composition are essential. There are now many tools available for this purpose^16^. In an earlier study, we selected gene sets to quantify immunological parameters in the TME by comparing them with similar gene sets available in the literature^17^. However, there were some discrepancies between the quantification results of cell populations by transcriptomic analyses and the density of the corresponding cell type in a tissue. Therefore, in this study, we applied several computational transcriptome analysis methods for evaluating the immune cell composition and immune-related TME of gastric cancer and compared the results to IHC and FCM data, which are regarded as gold standards for TME analysis. Transcriptome-based quantification of CD8^+^ T cells, regulatory T cells and B cells in the tumor was quite reliable (Fig. 1). Furthermore, we demonstrated that even spatial and functional analysis is feasible by RNA-Seq (Fig. 2); the “Exclusion score” was validated by the absence of CD8^+^ T cells in the core of the tumor as shown by IHC. The “Dysfunction score” was validated by the detection of fewer IFN-γ producing capacity of CD8^+^ T cells in the tumor using FCM. We also identified appropriate gene sets that reflected cell proliferation or cell death in the tumor (Fig. 3).

The output scores of the different methods allow either inter-sample comparisons of the same cell type, intra-sample comparisons between different cell types, or both (Table 2). In fact, IHC using a single antibody allows inter-sample comparisons, while FCM is good for intra-sample comparisons, depending on the panel of antibodies used. Therefore, transcriptome-based cell type quantification methods for inter-sample comparison were validated with IHC, and methods for intra-sample comparison were compared to FCM (Fig. 1). QuanTIseq, EPIC, and the absolute mode of CIBERSORTx generate an absolute score that can be interpreted as a cell fraction. Therefore, they can be utilized for both inter- and intra-sample comparisons and are quite useful in this context. Cell-type-specific estimation in the TME using bulk tumor data is challenging. Predicting transcriptionally distinct cell types would likely show good correlations. For example, Bindea et al. and Danaher et al. took FOXP3 as a single marker gene for Treg and successfully hit the best correlation coefficient of 0.6373 (Fig. 1). However, gene expression levels change according to their activation and differentiation status in many other cell types. Therefore, the gene set approach was used to address these complex problems as the wisdom of the crowd^18^. Although different gene sets were proposed by different methods, strategies to assemble gene sets for immune cells, in general, depend on the expression profiles of purified cell types to identify reference genes and therefore rely heavily on the data source from which the references are inferred and could this be inclined to overfitting these data. Therefore, it is difficult to cover the estimation of all cell types with a single prediction tool (Supplementary Fig. S3).

The difficulties in quantifying NK cells and macrophages are not limited to transcriptome-based methods. Because NK cells and macrophages express different arrays of cell surface receptors, the expression of which overlap in different cell populations, identification and quantification of these cells is not possible by a single marker assay^19,20^. For example, macrophage marker CD68 is also expressed by γδ T cells, NK cells, a subset of B cells, fibroblasts, and endothelial cells. CD163 is also expressed by dendritic cells. CD204 is expressed by only a subset of M2 macrophages. NKp46 is expressed by CD56^bright^ NK cells, but not CD56^dim^ NK cells^19^. Ki67 staining is used as a standard for evaluating proliferating cells. However, Ki67 protein levels were not a simple on-and-off switch of cell proliferation. Because Ki67 protein is continuously produced from the start of S phase and Ki67 is continuously degraded during G0 and G1 phase, quiescent cells re-entering the cell cycle will have varying levels of Ki67^21^. The discrepancy between transcriptome-based methods and IHC for NK cells, macrophages and cell proliferation might be due to the uncertainty of IHC results rather than the ambiguity of transcriptome-based methods. Multiplex IHC will overcome this problem and can be used as the gold standard for such assays.

IHC and FCM have been used as gold standards to estimate the immune cell content within a sample^10^. However, there are several limitations to these methods. Only a limited number of cell-type-specific markers can be utilized. FCM requires a large amount of sample that should be mechanically or enzymatically dissociated to isolate single-cell suspensions. Different single-cell dissociation efficiencies might bias the apparent proportions of immune cells in the tumor. In contrast, gene expression profiling by RNA-Seq provides comprehensive transcriptomics datasets derived from small tumor samples, and a large number of markers can be analyzed simultaneously. In addition to inflammatory molecules, many biological processes that shape the TME, such as angiogenesis, metabolism, and response to hypoxia, can be assessed and incorporated into the marker panel. Although we incorporated only 7 parameters that were validated by IHC or FCM into the TME analysis in this study, transcriptome-based TME analysis can easily be integrated with other molecular analyses and extended in the future.

Using 7 parameters extracted from RNA-Seq of bulk tissues, gastric cancers were immunologically classified into 3 clusters (Fig. 4). As reported previously, patients whose tumors had an immunologically “hot” TME had a better post-surgical prognosis^8^. Similar results were obtained in the current study (Fig. 4), although different gene sets and algorithms were applied. In Fig. 4a, two MSI cases, BKT008 and 038, were classified as belonging to the IP group, while the other 3 MSI cases were clustered in the IR group. The prognosis of these two IP patients was poor; BKT008 died of the primary disease 741 days after surgery and BKT038 after 347 days (Supplementary Tables S1 and S13). A poor immune response in the tumor might be responsible for the shorter OS of these two patients, despite their MSI subtype. In addition, BKT053, which was classified as CIN by TCGA classification and Hot1 by immunogram classification, was re-classified as IPp in this study. BKT053 relapsed on day 223 and died on 482 days. As shown in Supplementary Fig. S6a, transcriptome-based TME classification can discriminate IP patients from Hot1 patients in the TCGA cohort. Immunogram classification was based on the concept of the cancer-immunity cycle^7^, and TME classification was more associated with intratumoral immune response. Both methods are not mutually exclusive. Combining these two classifications can fine-tune the immunological subtypes of gastric cancer.

In conclusion, computational methods for transcriptomic analysis were validated by comparison with IHC and FCM to evaluate the TME of gastric cancer. It is feasible to evaluate the TME using RNA-Seq data obtained from small bulk tissues. For intra-sample comparison, either quanTIseq or CIBERSORTx is an appropriate tool to evaluate the immune cell fractions in TME. For inter-sample comparison, selecting the best score method for each parameter is better than any single method. Using 7 parameters selected, the TME of gastric cancer could be appropriately immunologically classified.

## Methods

### Patients and data sets

We enrolled 30 patients who underwent gastrectomy at Tokyo Metropolitan Bokutoh Hospital between June 2014 and October 2017 (Table 1). Clinical profiles with histology by the Lauren classification, overexpression of human epidermal growth factor receptor 2 (HER2) protein and the presence or absence of *Helicobacter pylori* infection were reported in our previous work^8^. In the previous study, RNA-Seq of bulk tumor tissue was performed^8^. RNA-Seq data of BKT patients are available at DDBJ Sequence Read Archive (Accession no. DRA009379)^8^. Clinicopathological features and RNA-Seq data for 375 additional gastric cancer patients were downloaded from the TCGA portal site (https://portal.gdc.cancer.gov/).

### Computational methods to analyze RNA-Seq data

RNA-Seq data were analyzed using the following algorithms or web tools: MCP-counter ("MCPcounter" R package)^22^, xCell (https://xcell.ucsf.edu/)^23^, TIMER (http://timer.cistrome.org/)^24^, quanTlseq (https://icbi.i-med.ac.at/software/quantiseq/doc/)^25^, EPIC (http://epic.gfellerlab.org)^26^, and CIBERSORTx (https://cibersortx.stanford.edu/)^27^. ssGSEA was performed using the gene sets of Bindea^28^, Davoli^29^, Danaher^30^, Cassetta^13^ and Consensus TME^31^ to enumerate immune cell composition. The enrichment scores were obtained using the ssGSEA method with R package ssGSEA 2.0 (https://github.com/broadinstitute/ssGSEA2.0) and R software version 3.6.0. The list of genes used by each method is provided in Supplementary Table S14. Because of the variation in the degree of specificity to which cell subsets were defined, summations of subsets were required to allow accurate comparisons in each cell type (Supplementary Tables S15 and S16). T cell dysfunction and exclusion scores were calculated on the TIDE website (http://tide.dfci.harvard.edu/)^14^. TCGA molecular classification of gastric cancer was performed as previously reported^6,8^. Gastric cancers were classified as EBV, MSI, genomic stability (GS), or chromosomal instability (CIN). Gastric cancers were also grouped as Mesenchymal or Non‐Mesenchymal subtypes by their 71‐gene mesenchymal signature according to the ACRG project^32^. Immunological subtypes based on immunogram scores were determined in our previous work^8^.

### Histological analysis

FFPE blocks were obtained from the Pathology Department of Tokyo Metropolitan Bokutoh Hospital. Immunohistochemistry was performed using the Ventana BenchMark automated immunostainer (Ventana Medical Systems, Tucson, AZ, USA) with labeled streptavidin–biotin and visualized with 3,3′-diaminobenzidine. The primary antibodies used were anti-CD3 (clone LN10, Leica), -CD4 (clone SP35, Ventana), -CD8 (clone 4B11, Leica), -CD45RO (clone UCHL-1, Ventana), -FOXP3 (clone 236A/E7, Abcam), -CD20 (clone L26, Leica), -NKp46 (clone #195314, R&D), -CD68 (clone Kp-1, Dako), -CD163 (clone 10D6, Leica), -CD204 (clone SRA-E5, Transgenic), -Ki-67 (clone MIB-1, Dako), -PD-L1 (clone E1L3N, Cell Signaling), -MLH1 (clone ES05, Leica), -MSH2 (clone FE11, Dako), -MSH6 (clone Polyclonal (Rabbit), GeneTex) and -PMS2 (clone M0R4G, Leica). EBV-encoded small RNA in situ hybridization (EBER-ISH) was performed on paraffin sections using a fluorescein isothiocyanate (FITC)-labeled peptide nucleic acid probe (Y5200; Dako, Glostrup, Denmark) and anti-FITC antibody (V0403, Dako). Slides were digitized with a Nanozoomer 2.0-HT virtual slide scanner (Hamamatsu Photonics, Hamamatsu, Japan) and observed in the NDP.view2 software (Hamamatsu Photonics). The density of immune cells was analyzed by Tissue Studio 2.0 software (Definiens, Munich, Germany).

### Flow cytometry

Tumors were cut into small pieces and enzymatically dissociated using a tumor dissociation kit (Miltenyi Biotec Inc., Auburn, CA, USA) to prepare fresh tumor digest (FTD) according to the manufacturer's instructions. After passing through a 70‐μm cell strainer (Thermo Fisher Scientific, Hampton, NH, USA), FTDs were cryopreserved in Bambanker™ freezing medium (NIPPON Genetics, Tokyo, Japan) until analysis. Cryopreserved FTDs were thawed in RPMI, and then stained using a Zombie Aqua™ Fixable Viability Kit (BioLegend, San Diego, CA, USA) with anti‐CD45 (clone 2D1, BioLegend) and -CD3 (clone HIT3a, BioLegend), -CD4 (clone SK3, Thermo Fisher Scientific), -CD8 (clone, HIT8a, BioLegend), -CD14 (clone: M5E2, BioLegend), -CD19 (clone J3-119, Beckman Coulter), -CD56-PE (clone N901, Beckman Coulter), -IFN‐γ (clone 45.15, Beckman Coulter, Brea, CA, USA), -TNF‐α (clone MAb11, BioLegend), -IL‐2 (clone MQ1-17H12, BioLegend). For the detection of cytokine production, cells were stimulated with 10 ng/ml Phorbol 12‐Myristate 13‐Acetate (PMA; Sigma‐Aldrich, St. Louis, MO, USA) together with 1 μg/ml Ionomycin (IM; Sigma‐Aldrich) or CytoStim (CS; Miltenyi Biotec) in the presence of 10 µg/ml brefeldin A (Sigma‐Aldrich) at 37ºC for 4 h. Intracellular cytokine staining was then carried out according to the manufacturer's instructions (using IntraPrep Permeabilization Reagent; Beckman Coulter). Stained cells were analyzed on a Gallios flow cytometer (Beckman Coulter) and data were processed using Kaluza (Beckman Coulter) and FlowJo (version 7.6.5; TreeStar, Ashland, OR, USA) software.

### Statistical analyses

For analyzing the correlation between each factor, Pearson's correlation coefficient method was used. For categorical variables, the chi-square test was used. A heat map was created using Ward’s hierarchical cluster analysis. The Kaplan–Meier method was used for survival analysis, and comparisons between groups were performed by log-rank testing. JMP Pro 15 (SAS Institute Japan, Tokyo, Japan) was used for statistical analysis. A value of *P* < 0.05 was considered statistically significant.

### Ethical declarations

This study was approved by the Research Ethics Committees of the University of Tokyo (No. G3545) and Tokyo Metropolitan Bokutoh Hospital (No. 25‐38‐02). All procedures followed were in accordance with the ethical standards of the responsible committee on human experimentation (institutional and national) and with the Helsinki Declaration of 1964 and later versions. Informed written consent was obtained from all patients included in the study.

## Supplementary Information

Supplementary Figures.

Supplementary Tables.

## Data Availability

Data are deposited on DDBJ Sequence Read Archive (Accession no. DRA009379).
