# Supplementary material for: Selection of RNA-based evaluation methods for tumor microenvironment by comparing with histochemical and flow cytometric analyses in gastric cancer
Source: Sci Rep. 2022 May 20;12:8576. doi: 10.1038/s41598-022-12610-w (PMC9122932; doi:10.1038/s41598-022-12610-w)
Supplement: Supplementary file 1 — Supplementary Figures. [file 41598_2022_12610_MOESM1_ESM.pdf]

Supplementary Information
（Figures）

Selection of RNA-based evaluation methods for tumor microenvironment by comparing bulk RNA-seq bioinformatics tools with histochemical and flow cytometric analyses in gastric cancer

Noriyuki Saito, Yasuyoshi Sato, Hiroyuki Abe, Ikuo Wada, Yukari Kobayashi, Koji Nagaoka, Yoshihiro Kushihara, Tetsuo Ushiku, Yasuyuki Seto, Kazuhiro Kakimi,

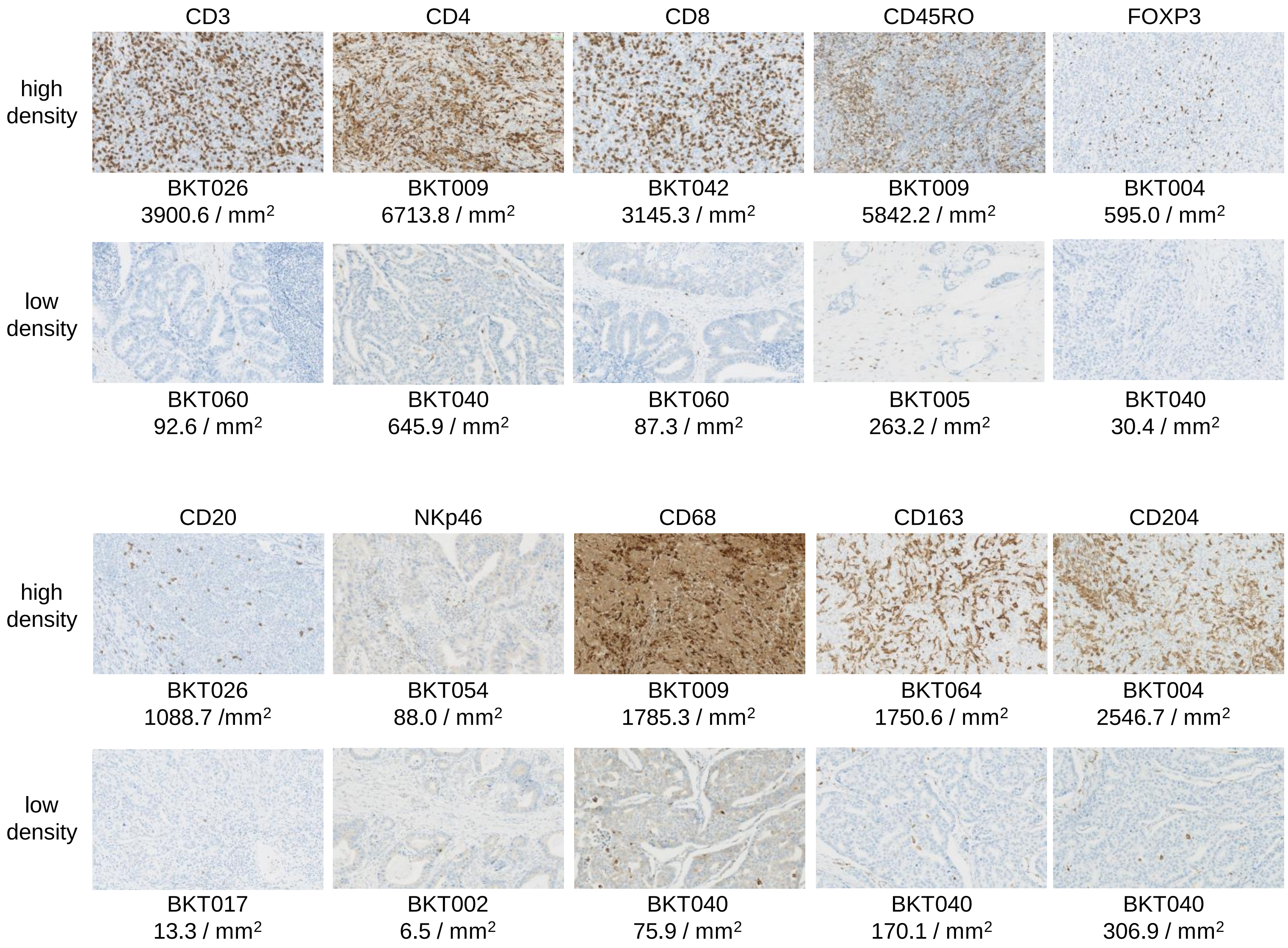

Supplementary Figure S1. Immune cell density measurement by IHC

The staining patterns of CD3, CD4, CD8, CD45RO, FOXP3, CD20, NKp46, CD68, CD163 and CD204 are depicted. To obtain the cell densities, the number of positive cells was divided by tumor tissue area (mm^2). The slides with the highest and the lowest densities in each antibody are displayed.

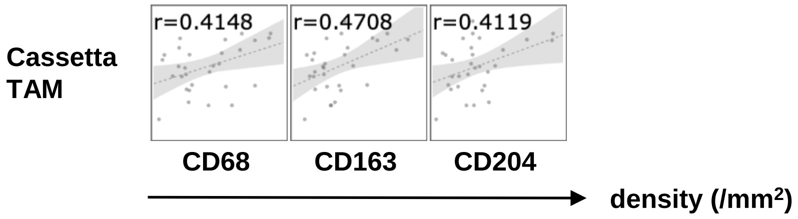

Supplementary Figure S2. Correlation between transcriptome-based quantification methods and IHC The Pearson‘s correlation coefficients (r) between ssGSEA score using Cassetta’s gene set for TAM and the densities of immune cells detected by the indicated antibodies (/mm^2) are shown in the upper part of each scatter plot.

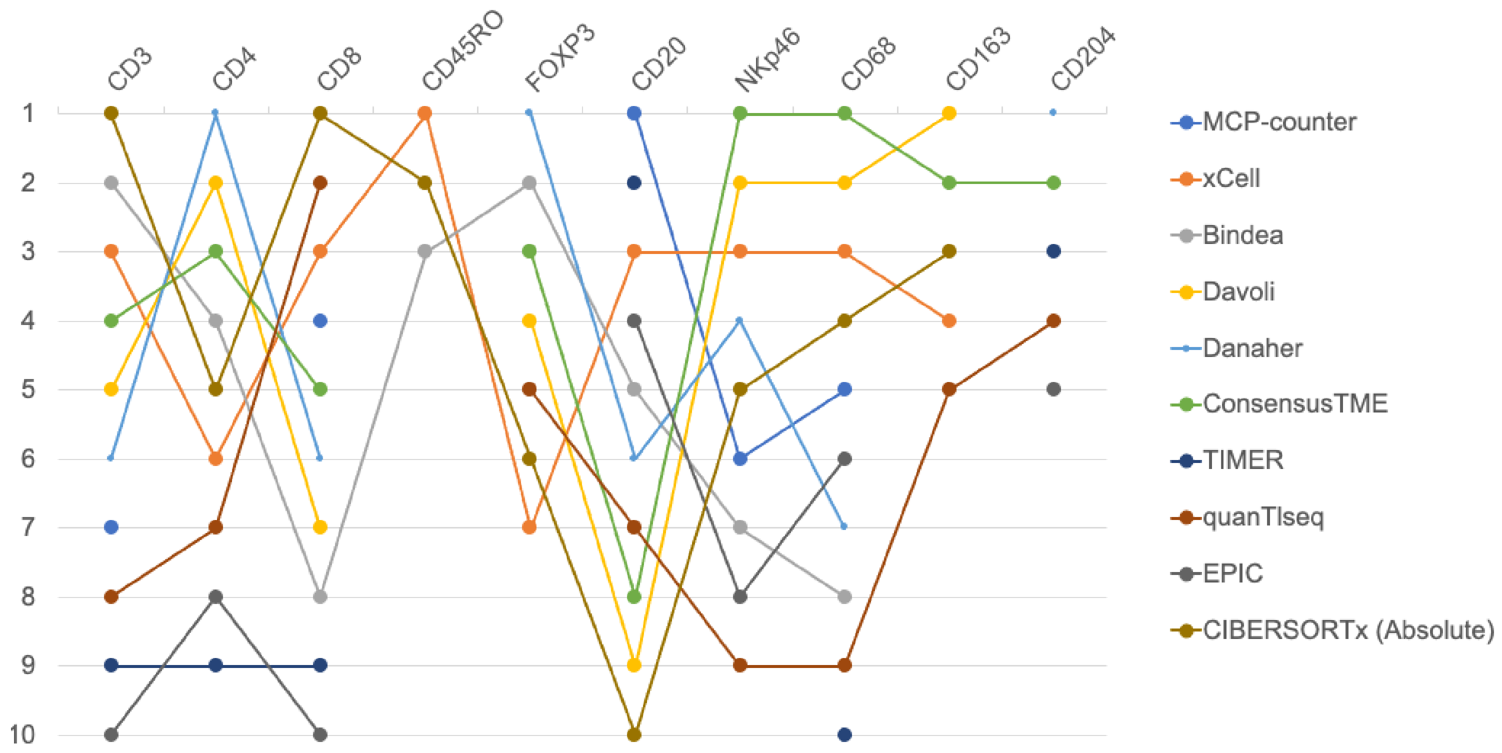

Supplementary Figure S3. The rank of TME cell estimation methods across various immune cells Transcriptome-based methods were ranked based on their correlation coefficient with the densities of cells by immunohistochemistry.

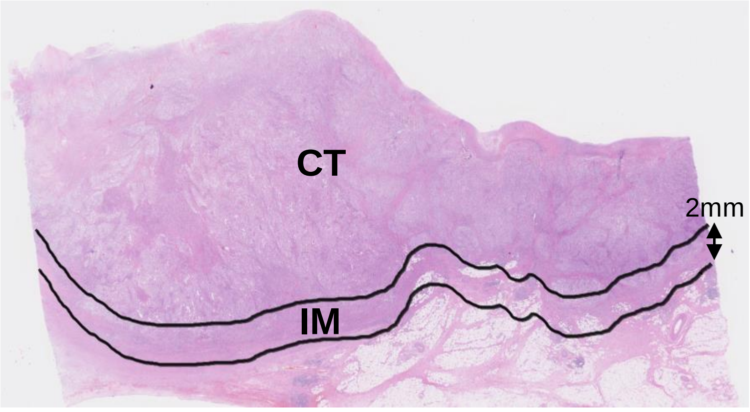

Supplementary Figure S4. Spatial analysis of tumor-infiltrating cells

The tumor tissue was divided into the core of the tumor (CT) and the invasive margin (IM). The invasive margin was defined as the region 1 mm in width on each side of the border between malignant cells and normal tissue. The density of immune cells was measured in CT and IM and the the ratio of cell densities between CT and IM was calculated.

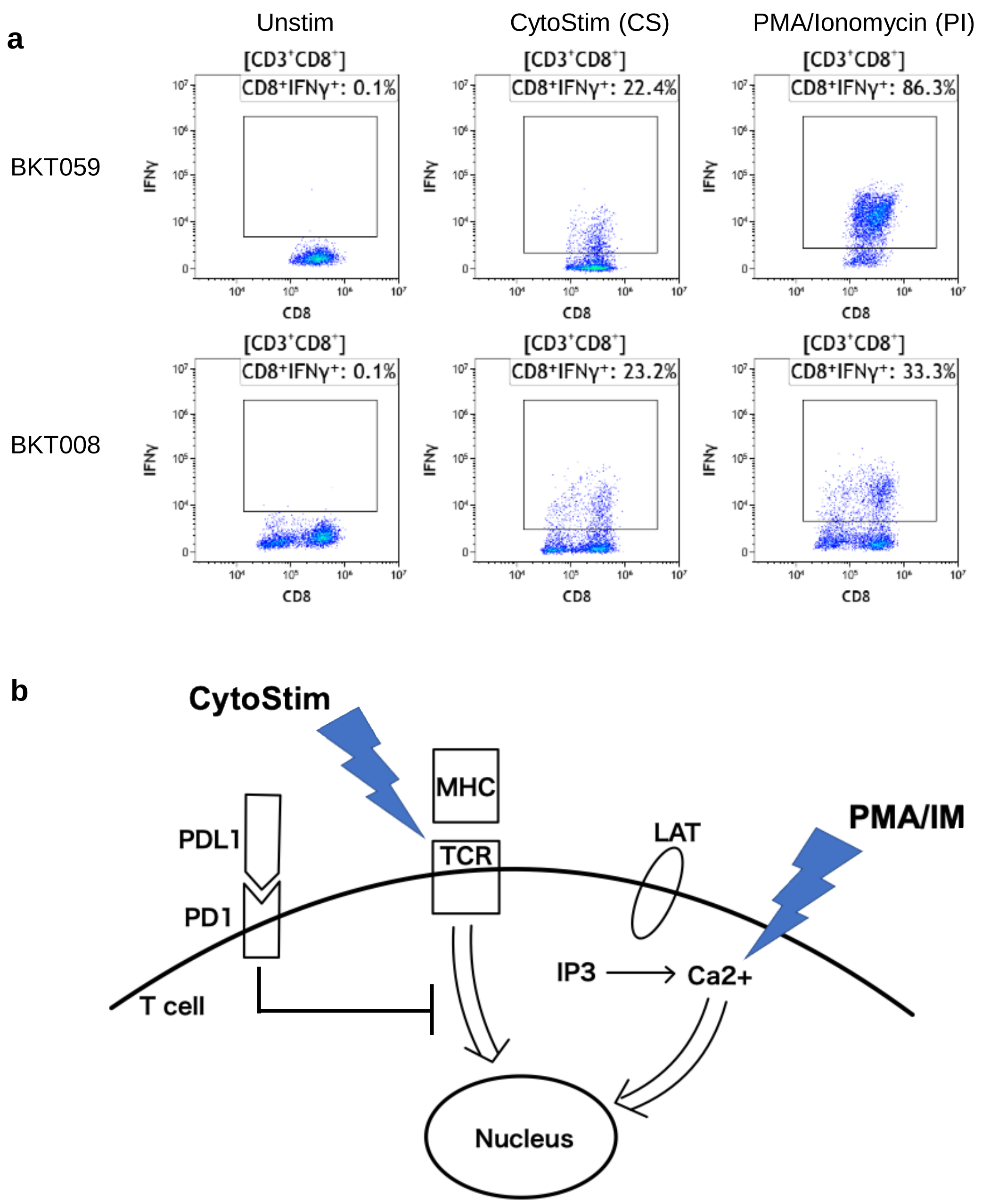

Supplementary Figure S5. Functional analysis of tumor-infiltrating T cells

(a) The cytokine-producing capacity of tumor-infiltrating T cells was evaluated by intracellular cytokine staining. Tumor-infiltrating cells were left untreated (Unstim) or stimulated with CytoStim (CS) or PMA/IM (PI) for 4 h. The cells were then stained with the indicated antibodies and evaluated by flow cytometry. (b) TCR signaling. CytoStim stimulates MHC-TCR interaction, while PMA/ionomycin increases intracellular Ca^{2+} concentration, stimulating T cells and inducing cytokine secretion.

a

Immunogran	TME	Immune-Rich (IR)	Immune-Poor dysfunctional (IPd)	Immune-Poor proliferative (Ipp)
	n	178	69	128
**Hot1**	80	61 (76.3)	10 (12.5)	9 (11.3)
**Hot2**	11	9 (81.8)	2 (18.2)	0 (0.0)
**Intermediate**	97	74 (76.3)	19 (19.6)	4 (4.1)
**Cold**	187	34 (18.2)	38 (20.3)	115 (61.5)

b

c

d

e

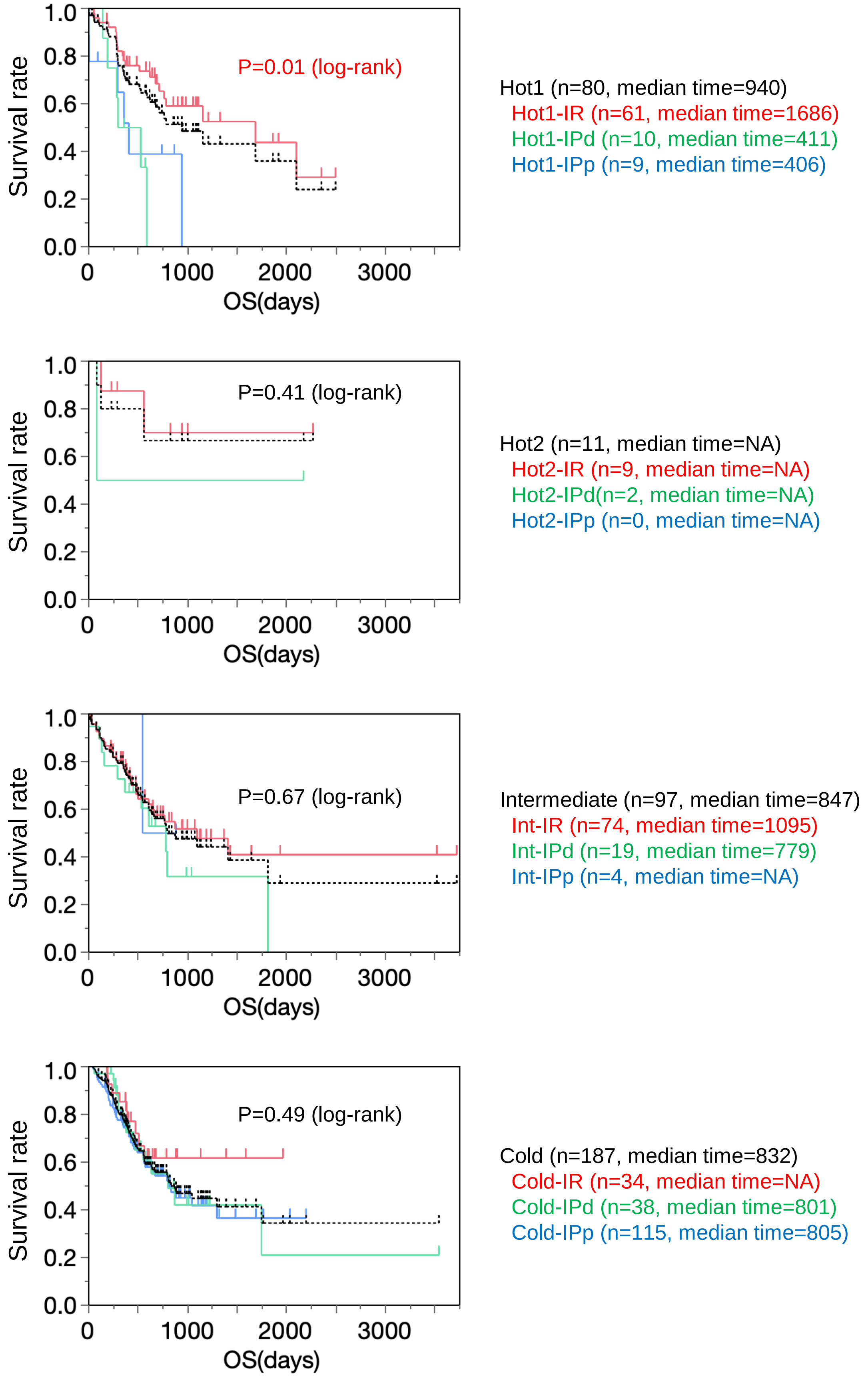

Supplementary Figure S6. Comparison of immunogram classification and transcriptome-based TME classification in TCGA cohort (a) Re-classification of gastric cancer patients by transcriptome-based TME classification for immunogram classified TCGA cohort (b) The Kaplan-Meier method and log-rank test were performed for the survival analysis of Immune-Rich (IR, red) Immune-Poor dysfunctional (IPd, green) and Immune-Poor proliferative (IPp, blue) groups in Hot1 patients. The entire survival curve of Hot1 patients was depicted in a dashed black line. Similarly, survival analyses for Hot2 (c), intermediate (d) and cold (e) patients were performed.
